# The first coronary by-pass grafting surgery done in western and central Africa

**DOI:** 10.4314/pamj.v8i1.71163

**Published:** 2011-04-24

**Authors:** Appolonia Budzee, Italo Ghidoni, Alessandro Giamberti, Silvia Cirri, Jacques Cabral Tantchou Tchoumi, Jean Claude Ambassa, Gianfranco Butera

**Affiliations:** 1 St. Elizabeth Catholic General hospital, cardiac centre P.O. Box 8 Kumbo, Cameroon; 2 Hesperia Hospital Modena, Italy; 3 Policlinico San Donato IRCCS, 20097 San Donato, Milanese, Italy

**Keywords:** Coronary artery bypass grafting, cardiac centre, Cameroon

## Abstract

Africa bears a significant proportion of the global burden of chronic diseases, along with poor countries of Asia and Latin America. The World Health Organisation projects that over the next ten years the Continent will experience the largest increase in death rates from cardiovascular disease, cancer, respiratory disease and diabetes. Probably for the first time in Western and Central Africa, the very first coronary artery bypass surgery grafting was performed in the Cardiac Centre of Shisong in Cameroon.

## To the editors of the Pan African Medical Journal

As a result of progressive urbanization and westernization of their lifestyle, developing Countries are now undergoing an epidemiological transition. These changes are leading to a new epidemiological situation in the world with a decline in infectious diseases and emergence of cardiovascular diseases in general and coronary artery disease in particular [1]. Studies in the 1970s and 1980s suggested that the prevalence and death rates from coronary artery disease were low in the black African population. However, many reports from different African countries suggest that the spectrum and pattern of cardiovascular diseases along with their risk factors are changing rapidly, particularly in urban areas [2,3].

For the first time in Central Africa and in Sub-Saharan Africa (South Africa excluded) two cases of coronary artery bypass surgery grafting were performed in the Cardiac Centre of Shisong in Cameroon. Coronary artery bypass grafting (CABG) surgery is only used to treat people who have severe coronary artery disease that could lead to a heart attack. CABG can be recommended if other treatments, such as lifestyle changes or medicines, haven’t worked. A CABG may also be recommended if there are severe blockages in the large coronary arteries that supply a major part of the heart muscle with blood especially if the heart’s pumping action has been already weakened. CABG also may be a treatment option if you have blockages in the heart that can’t be treated with angioplasty.

**Figure 1:**
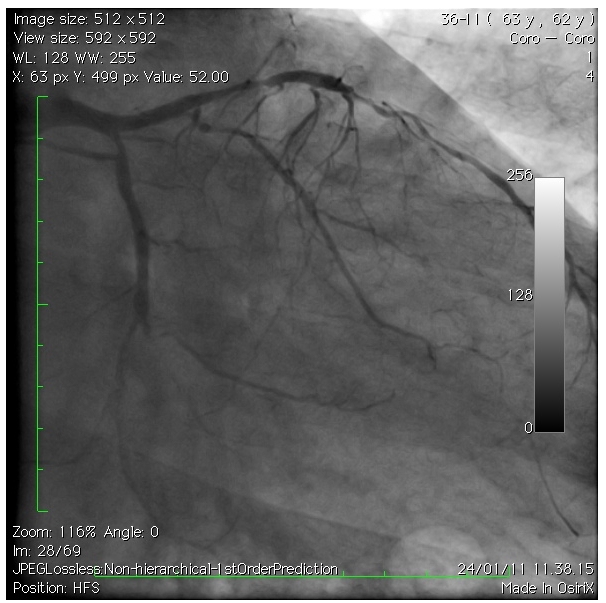
Left anterior oblique view with cranial angulation showing multiple and critical coronary artery stenosis

Mr A, one of those who benefited from the surgery is an obese hypertensive patient having shortness of breath in very mild physical exertion, with retrosternal chest pains without irradiation. No documented history of myocardial infarction, but the patient remembered some episodes of severe chest pains relieved spontaneously few years ago. The ECG done was showing an aspect of antero septal and apical scar, the cardiac echo was showing a poor global contractility with hypokinetic left ventricular apex, interventricular septum. The diagnosis of ischemic cardiomyopathy was done and a coronarography requested. On the angiography, we found a tritroncular stenosis of more than 75% (Figure 1). The coronary bypass grafting procedure was indicated. The surgery was performed on February 10 2011 with success. The patient was transferred to the intensive care unit where his two days of stay were without event. He was then transferred to the ward and discharged a week after.

“I’ve been moving from hospitals to hospitals without improvement. Finally I’m very happy that my problem has been discovered and solved” said Mr A going home and already able to perform some exercises almost impossible before surgery.

The Cardiac Center offers a great opportunity to forestall the upsurge of occurrence of cardiovascular diseases in the Region; however, financial constraints hamper patients’ ability to access it. We are prepared to break through a system where lack of solidarity seriously undermines the underprivileged and blinds the rich; a social system which fails to consider the unemployed, the under employed and those without access to health insurance; a system that weakens rather than strengthen the needy; a system where cardiac and other patients are abandoned to their fate. Planted in the 75 year old St. Elizabeth Catholic Hospital, the Shisong Cardiac Centre is the fruit of efforts between the Tertiary Sisters of St Francis, Cuore Fratello and Bambini Cardiopatici nel Mondo Association of San Donato in Italy.

This is the only cardio-surgical Center in Central / West Africa, equipped with ultra modern health technologies and prepared to offer diverse services; such as cardiology, including diagnosis and treatment of congenital heart defects, coronary artery disease, valvular heart disease and electrophysiology. It manages non-invasive cardiology; that is, diagnostic testing for patients with suspected cardiac problems through tests such as electrocardiography, holter, stress test, three-dimensional, color, pulsed and continuous Doppler-echocardiography. It also offers both diagnostic and interventional catheterism in a heamodynamic laboratory, implantation of permanent mono, bicameral pace makers and defibrillators also open-heart surgeries with extracorporeal circulation.

Numerous surgical teams travel to underdeveloped countries to perform surgery each year and train the local surgeon and ancillary personnel as best they can. However, in most such cases, the surgical teams are present at those sites for no more than one week per year, leaving the local population and surgeons to struggle themselves for the remainder of the year [4]. The World Heart Foundation proposes that rather than continuing with the current disorganized and inefficient system, the problem should be addressed by multidimensional approaches directed at both an improvement in surgical services and an enhancement of education and training, taking maximum advantage of contemporary communications technology and educational techniques.

Thanks to the dynamic skill in organization and management of the executive board, cardio-surgical missions have been going on monthly for 16 months since the inauguration of the Centre in November 2009 [4]. One hundred and fifty cases have been operated with extracorporeal circulation, ten pacemakers have been implanted, interventional and diagnostic catheterizations were performed in 85 patients.

The first coronary artery bypass surgery grafting is opening a new page in the surgical management of coronary artery disease in Cameroon. In a context of financial limitation, government and development partners support is critical; the educational value of such structure ought to be exploited by health professional and students in the Country.

## Competing interests

The authors are all affiliated to the Shisong Cardiac Centre, Cameroon.
